# Congenital Diaphragmatic Hernia Requiring ECMO: Current Evidence, Controversies, and Unresolved Questions

**DOI:** 10.3390/children13101417

**Published:** 2026-10-15

**Authors:** Africa Pertierra Cortada, Cristina Carrasco Carrasco

**Affiliations:** 1 Neonatology Department, BCNatal—Centre de Medicina Maternofetal i Neonatologia de Barcelona, Hospital Sant Joan de Déu, Universitat de Barcelona, 08950 Barcelona, Spain; 2 Institut de Recerca Sant Joan de Déu, 08950 Barcelona, Spain; cristina.carrasco@sjd.es

**Keywords:** congenital diaphragmatic hernia, extracorporeal membrane oxygenation, ECMO candidacy, veno-arterial ECMO, veno-venous ECMO, surgical timing, surgical repair, cannulation, ECMO complications, ECMO outcomes

## Abstract

**Highlights:**

**What are the main findings?**

- No single parameter or protocol reliably guides ECMO candidacy, mode, timing of repair, or duration in CDH.
- Postnatal echocardiographic assessment of ventricular dysfunction and pulmonary hypertension is key to risk stratification, and recent observational analyses suggest a potential benefit of early repair during ECMO in selected patients.

**What are the implications of the main finding?**

- CDH-ECMO management should be individualized, integrating cardiopulmonary status rather than fixed thresholds.
- Prospective multicenter studies are needed to standardize definitions and reduce practice variation.

**Abstract:**

**Background/Objectives:** Congenital diaphragmatic hernia (CDH) requiring extracorporeal membrane oxygenation (ECMO) remains one of the most challenging conditions in neonatal critical care. Despite advances in prenatal assessment and neonatal management, substantial uncertainty persists regarding ECMO selection, mode and duration of support, and timing of diaphragmatic repair. This review summarizes the available evidence and critically examines the major unresolved questions in the management of infants with CDH requiring ECMO. **Methods:** A narrative review was conducted based on a literature search in PubMed/MEDLINE, Scopus, Web of Science, and the Cochrane Library for studies published between June 2005 and June 2026. Additional relevant publications were identified through reference screening, and selected foundational studies predating the search period were included when appropriate. Prospective and retrospective studies, registry-based analyses, systematic reviews, meta-analyses, and relevant consensus statements were considered. Evidence was narratively synthesized, with particular attention to disease severity, methodological limitations, and survivor bias. **Results:** Available evidence highlights substantial heterogeneity in disease severity and management. Prenatal and postnatal markers aid risk stratification, but no single parameter reliably predicts ECMO requirement or defines the optimal management strategy. To date, no clear survival advantage has been demonstrated for either veno-arterial or veno-venous ECMO, although veno-arterial ECMO remains the predominant mode of support in most centers; mode selection should therefore be individualized according to cardiopulmonary physiology, patient characteristics, anatomy, and institutional expertise. Surgical timing remains particularly controversial. While repair after ECMO decannulation has been associated with favorable outcomes, recent observational analyses suggest that early repair during ECMO may be beneficial in selected patients, although the optimal timing remains uncertain. Prolonged ECMO may provide additional time for pulmonary and cardiovascular recovery, although this potential benefit must be weighed against the cumulative morbidity of prolonged support. **Conclusions:** Management should be individualized according to disease severity, clinical trajectory, cardiopulmonary recovery, and institutional expertise. Current evidence does not support a universal strategy for ECMO initiation, mode, surgical timing, or duration. Prospective multicenter studies using standardized definitions and adequately accounting for disease severity are needed to reduce practice variation and better define optimal management.

## 1. Introduction

Congenital diaphragmatic hernia (CDH) is a congenital anomaly characterized by a defect in the diaphragm that permits herniation of abdominal viscera into the thoracic cavity [1]. The condition is associated with abnormal pulmonary and cardiovascular development, which largely determines postnatal outcome. Although its etiology remains incompletely understood, current evidence supports a multifactorial pathogenesis involving both genetic susceptibility and environmental influences. According to the widely accepted dual-hit hypothesis [2,3,4], an early developmental insult affects pulmonary development bilaterally before diaphragmatic closure, whereas herniation of the abdominal viscera subsequently exacerbates ipsilateral pulmonary hypoplasia by restricting thoracic growth and interfering with normal fetal respiratory movements [5]. Together, these mechanisms result in the characteristic pathophysiological triad of pulmonary hypoplasia, pulmonary hypertension, and cardiac dysfunction [2,6,7].

Pulmonary hypoplasia is characterized by impaired airway and alveolar development, leading to reduced functional lung volume and severe respiratory insufficiency [6]. In parallel, abnormal pulmonary vascular remodeling contributes to persistent pulmonary hypertension [8], increased right ventricular afterload, and impaired postnatal cardiopulmonary adaptation [9]. Cardiac dysfunction represents the third major component of CDH pathophysiology [10,11,12]. Altered fetal cardiac loading conditions, together with mechanical compression and reduced pulmonary venous return, impair left ventricular growth and contribute to postnatal ventricular dysfunction. The interaction among these abnormalities, together with associated congenital anomalies, gestational age, and birth weight, accounts for the broad spectrum of clinical severity observed in infants with CDH.

Despite significant advances in prenatal diagnosis, neonatal intensive care, and surgical management, CDH remains associated with considerable morbidity and an overall mortality of approximately 20–30% [13,14]. In the most severe cases, extracorporeal membrane oxygenation (ECMO) provides temporary cardiorespiratory support while allowing time for pulmonary and cardiovascular adaptation. However, several aspects of ECMO management remain controversial, including patient selection, the choice of ECMO modality, the optimal timing of diaphragmatic repair, and the appropriate duration of extracorporeal support. Consequently, management strategies continue to vary considerably among institutions. In this review, we summarize the current evidence and discuss the major controversies surrounding the management of infants with CDH requiring ECMO support.

## 2. Methods

### 2.1. Review Design and Rationale

From the outset, this article was conceived as a narrative, non-systematic author review rather than a systematic review. This design was chosen because the evidence on ECMO in congenital diaphragmatic hernia (CDH) consists predominantly of retrospective single-center series and registry-based analyses, with few prospective studies, and no randomized controlled trial has addressed ECMO candidacy, ECMO mode, timing of diaphragmatic repair, or duration of extracorporeal support in this population. The available studies are also highly heterogeneous in study period, patient population (e.g., CDH laterality, prenatal severity, prior fetal endoscopic tracheal occlusion), definitions (e.g., “early” versus “late” repair, “prolonged” ECMO), and outcome measures, which precludes meaningful quantitative synthesis. Our aim was therefore to provide a structured and critically appraised narrative synthesis of the available evidence, complemented, where evidence was lacking, by the authors’ clinical experience in a tertiary neonatal ECMO referral center.

### 2.2. Search Strategy and Study Selection

PubMed/MEDLINE, Scopus, Web of Science, and the Cochrane Library were searched for peer-reviewed articles published in English between June 2005 and June 2026 (last search: June 2026). Searches combined “congenital diaphragmatic hernia” and “extracorporeal membrane oxygenation” or “ECMO” with topic-specific terms (e.g., indications, complications, venovenous, venoarterial, cannulation, repair, and timing of surgery). The reference lists of key articles and current guidelines (ELSO, CDH EURO Consortium, and national CDH collaboratives) were also reviewed. In addition to the database searches, other studies considered important for the review because of their design or findings were included, some of them published before 2005. Clinical studies, registry analyses, systematic reviews, guidelines, and expert reviews addressing ECMO in CDH were included; case reports were included only for technical aspects not covered elsewhere. Conference abstracts and editorials were excluded. Each author screened the records retrieved by her own searches and selected the most relevant articles for the sections she drafted, giving priority to multicenter and registry-based studies, systematic reviews, and studies addressing disease severity or survivor bias. Consistent with the narrative design, record counts and a PRISMA flow diagram are not reported.

### 2.3. Evidence Synthesis

Of the 120 publications cited, 63 (53%) were retrospective, 8 were prospective studies, and none of the three randomized trials identified in this review specifically evaluated infants with CDH on ECMO [15,16,17]. No formal risk-of-bias assessment was performed. The evidence was synthesized narratively by topic; the main limitations of the available studies, such as differences in disease severity and survivor bias, are discussed in the text, and where evidence was insufficient, the interpretation reflects the authors’ clinical experience.

## 3. Discussion

### 3.1. Prediction of ECMO in Neonates with CDH

In general, anatomical, physiological, and clinical parameters associated with mortality also correlate with the need for extracorporeal membrane oxygenation (ECMO) in infants with congenital diaphragmatic hernia (CDH) [18]. Prenatally, the degree of pulmonary hypoplasia is a major determinant of postnatal disease severity and is associated with both mortality and ECMO requirement. Ultrasound-derived observed-to-expected lung-to-head ratio (O/E LHR) is widely used for prenatal risk stratification, with lower values associated with more severe pulmonary hypoplasia and a higher likelihood of requiring ECMO [19]. An O/E LHR < 25% identifies a particularly severe phenotype and was used as the inclusion criterion for severe left-sided CDH in the TOTAL trial evaluating fetal endoscopic tracheal occlusion (FETO) [15,20].

Fetal magnetic resonance imaging (MRI) provides a three-dimensional assessment of fetal lung volume and may offer additional prognostic information. Observed-to-expected total fetal lung volume (O/E TFLV) has been associated with postnatal outcomes, including ECMO requirement, and may complement ultrasound-based assessment of pulmonary hypoplasia [21]. More recent data further support the prognostic value of fetal MRI, with third-trimester percent predicted lung volume (PPLV) showing particularly strong discrimination for ECMO utilization. In a recent cohort, a late-gestation PPLV ≤ 17.6% was the strongest single imaging predictor of ECMO utilization in left-sided CDH, whereas a threshold of ≤16.4% showed the best discrimination in right-sided CDH [22]. However, differences in imaging methodology, gestational age, CDH laterality, and study populations limit the applicability of a single universal threshold. Prenatal lung volume assessment should therefore be regarded primarily as a tool for severity stratification and antenatal counseling rather than as a standalone criterion for determining the need for ECMO. Accordingly, in fetuses with the most severe pulmonary hypoplasia, defined by a PPLV < 15% on fetal MRI, the ex utero intrapartum treatment (EXIT)-to-ECMO procedure has been proposed to avoid hypoxemia and barotrauma before cannulation. However, evidence is limited to small single-center series. In the only comparative study, all infants with PPLV < 15% eventually required ECMO, yet survival was 33% (2/6) with EXIT-to-ECMO compared with 50% (5/10) with conventional delivery, showing no clear survival benefit [23].

Postnatally, echocardiographic assessment provides complementary information by characterizing the cardiovascular consequences of pulmonary hypertension [24]. Early ventricular dysfunction, particularly impaired left ventricular systolic function, is associated with greater disease severity and adverse outcomes. In a prospective observational study, left ventricular global longitudinal strain assessed during the first 48 h of life correlated with prenatal and postnatal markers of disease severity and was significantly lower in infants who died and/or required ECMO [25]. Thus, integration of prenatal assessment of pulmonary hypoplasia with postnatal evaluation of respiratory and cardiovascular function may allow a more comprehensive approach to identifying infants at high risk of requiring ECMO.

### 3.2. Patient Selection

Patient selection appears to be a critical determinant of the potential benefit derived from ECMO [18,26,27,28]. Morini et al. reported that the mortality benefit associated with ECMO was concentrated among infants deemed ECMO candidates, with reductions observed in both early mortality overall and, within the candidate subgroup, late mortality [26,28]. More recently, Jancelewicz et al. evaluated ECLS-eligible infants using propensity-score matching and found that the association between ECLS and improved survival was concentrated among patients at highest predicted risk, whereas a survival benefit was not observed in lower-risk groups [29]. These findings support a selective rather than universal approach to ECMO in CDH, emphasizing the importance of integrating disease severity, clinical trajectory, and potentially reversible cardiopulmonary dysfunction when considering ECMO initiation.

### 3.3. Indications of ECMO

ECMO should be considered in infants with CDH who develop severe, refractory cardiopulmonary failure despite optimized conventional management [19,27]. By providing respiratory and, when required, circulatory support, ECMO may allow time for pulmonary vascular resistance and ventricular function to improve while avoiding further escalation of ventilatory pressures [27]. Current consensus recommendations identify persistent hypoxemia, severe respiratory or metabolic acidosis, refractory hemodynamic instability despite optimized medical therapy, and the requirement for high ventilatory pressures as potential indications for ECMO [19,30]. Proposed physiological thresholds include inability to maintain preductal oxygen saturation > 85% or postductal oxygen saturation > 70%; respiratory acidosis with pH < 7.15 despite optimized ventilatory management; oxygenation index (OI) ≥ 40; and inadequate oxygen delivery with metabolic acidosis, particularly lactate ≥ 5 mmol/L [30] (Table 1).

However, these thresholds should not be regarded as absolute criteria, as no single physiological parameter has been prospectively validated to determine the optimal timing of ECMO initiation in infants with CDH. In particular, OI > 40 may not fully capture the complex interaction between pulmonary hypoplasia, pulmonary hypertension, ventricular dysfunction, and impaired systemic perfusion that characterizes severe CDH [19,31]. Therefore, ECMO candidacy should be based on the overall clinical trajectory of respiratory and cardiovascular failure and the response to optimized medical therapy, complemented by serial echocardiographic assessment of ventricular function and pulmonary hypertension [19,27].

Contraindications to ECMO are primarily related to conditions associated with an extremely poor prognosis or an unacceptably high risk of severe irreversible morbidity [19,27]. Absolute or near-absolute contraindications generally include lethal chromosomal or genetic abnormalities, severe irreversible neurological injury, major intracranial hemorrhage, uncontrolled bleeding, and complex or unrepairable cardiac anomalies. In a modified Delphi study conducted by the American Pediatric Surgical Association Critical Care Committee, all respondents considered complex or unrepairable cardiac defects and lethal chromosomal abnormalities contraindications to ECMO, while 94.1% considered severe intracranial hemorrhage a contraindication [32]. However, these findings represent expert consensus rather than prospective outcome-based evidence.

Other factors, including extreme prematurity, very low birth weight, prolonged mechanical ventilation before ECMO, and severe associated anomalies, should generally be considered relative rather than absolute contraindications and assessed on an individual basis [19,27,33]. Overall, ECMO candidacy should be determined by the expected likelihood of meaningful survival and long-term outcome rather than by isolated gestational age, birth weight, or physiological criteria.

### 3.4. Type and Mode of Support

To date, no clear survival advantage has been demonstrated for either veno-venous (VV) or veno-arterial (VA) ECMO in infants with CDH [19,34,35,36,37,38,39,40,41], although VA remains the predominant mode of support in most centers [36,37,39,42,43,44].

Both VA and VV ECMO offer distinct physiological advantages that may be beneficial in different clinical scenarios. VA ECMO provides both respiratory and circulatory support, making it the preferred modality for patients with severe hemodynamic compromise in addition to respiratory failure. By unloading the right ventricle and reducing myocardial workload, it may facilitate cardiovascular recovery in patients with persistently elevated pulmonary vascular resistance, a hallmark of CDH [18,19]. In addition, VA cannulation is often technically less demanding in neonates because it is less affected by the limitations imposed by small venous caliber and is generally less prone to flow instability or cannula malposition.

In contrast, VV ECMO provides respiratory support while preserving physiological circulation and pulsatile systemic blood flow. By allowing oxygenated blood to continue perfusing the pulmonary vasculature, it may promote pulmonary vasodilation and ensure the delivery of highly oxygenated blood to the left heart and coronary circulation. In addition, because systemic perfusion is maintained without retrograde arterial flow, VV ECMO preserves more physiological left ventricular loading conditions and avoids the increase in left ventricular afterload associated with VA ECMO, potentially reducing the risk of myocardial stunning. Furthermore, VV ECMO avoids carotid artery cannulation, thereby eliminating complications related to carotid ligation and potentially reducing the incidence of cerebral embolic and acute neurological events [18,19,36,45,46], although the evidence supporting this neurological benefit remains controversial [39,47].

An important limitation of VV ECMO is the potential need for conversion to VA support. Registry data [39] and observational studies [48] consistently show that infants requiring conversion experience higher mortality and an increased risk of neurological injury compared with those who remain on their initial ECMO modality, although accurately identifying patients who will ultimately require escalation remains challenging.

Overall, current evidence does not demonstrate the superiority of either VA or VV ECMO in infants with CDH. The absence of randomized comparative studies and the predominance of retrospective observational data continue to limit definitive recommendations. Therefore, the choice of ECMO configuration should be individualized according to the patient’s cardiorespiratory physiology, institutional expertise and preference, prior negative experience with VV, the anticipated risk of conversion, the patient’s size and vascular anatomy, and the availability of appropriate cannulation equipment [19,38,39,44].

### 3.5. Cannulation Strategies and Technical Considerations in CDH Patients

Although the choice of ECMO configuration is determined by the patient’s clinical condition, successful support ultimately depends on safe and accurate cannulation. In infants with CDH, mediastinal displacement and distortion of the thoracic venous anatomy frequently alter normal vascular relationships, making vascular access particularly challenging.

These anatomical constraints are particularly relevant during VV ECMO cannulation. In addition to mediastinal displacement, reduced internal jugular vein diameter has been described in infants with CDH, further limiting the feasibility of dual-lumen VV cannulation [49]. Moreover, the smallest commercially available dual-lumen cannula is 13 Fr, which may preclude its use in infants weighing less than 2.5 kg. In selected cases, the right brachiocephalic vein has been proposed as an alternative cervical access when internal jugular cannulation is not feasible [49]. Likewise, when dual-lumen cannulation cannot be achieved, a two-cannula VV configuration using the internal jugular vein for drainage and the femoral vein for reinfusion may represent a feasible alternative [50,51,52,53,54,55], although femoral vessel size and recirculation remain important limitations [56].

Because the performance of dual-lumen VV ECMO is highly dependent on accurate cannula positioning, real-time imaging guidance during cannula insertion is strongly recommended. Echocardiography, with or without fluoroscopy, facilitates precise placement of the cannula tip and helps minimize malposition and recirculation [50]. Beyond cannula insertion, echocardiography remains the imaging modality of choice for all ECMO configurations. It allows confirmation of optimal cannula position, assessment of cardiac function, and early detection of cannula displacement or other complications throughout the ECMO run [19,57,58,59,60].

**Figure 1:**
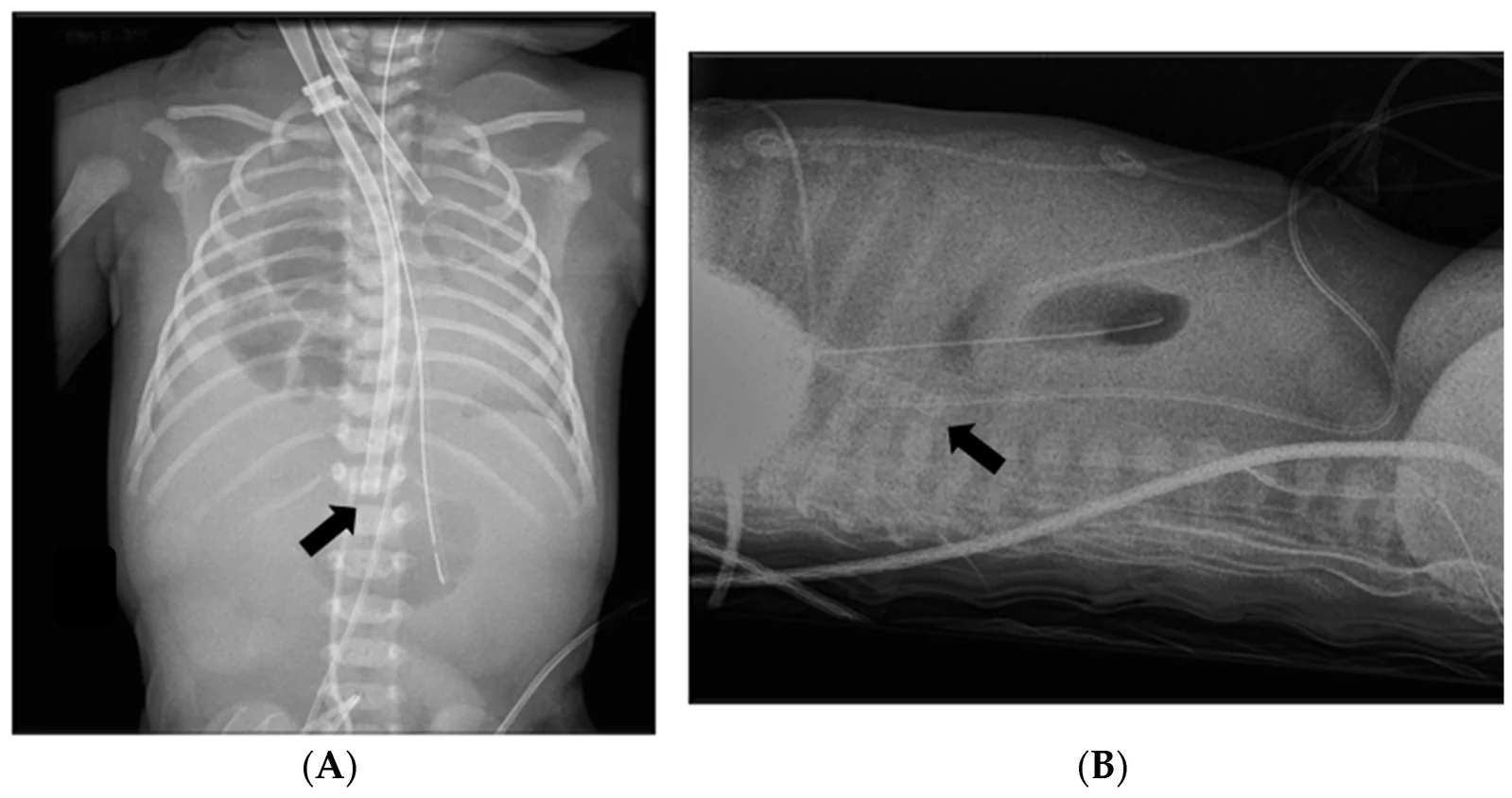
ECMO cannulation in a patient with right-sided CDH, showing deviation of the venous cannula toward the azygos vein: (**A**) Initial anteroposterior chest X-ray after cannulation, showing the venous cannula tip positioned well below the diaphragm (black arrow). (**B**) Lateral chest X-ray showing posterior deviation of the venous cannula behind the heart (black arrow), consistent with an azygos vein course. Authors’ own images.

Particular attention should be paid to infants with right-sided CDH. Liver herniation may compress the inferior vena cava, resulting in compensatory enlargement of the azygos vein and distortion of the normal thoracic venous anatomy. These changes increase the risk of inadvertent azygos vein cannulation [61,62] (Figure 1), which should be suspected in the presence of inadequate venous drainage or unexplained circuit dysfunction. Because anteroposterior chest radiography may appear reassuring despite malposition, echocardiography is essential to confirm that the venous cannula is correctly positioned within the superior vena cava or right atrium. When uncertainty persists, a lateral chest radiograph demonstrating a posterior course of the catheter strongly suggests azygos vein cannulation [49,61,62,63] (Figure 1). Overall, meticulous preprocedural planning together with systematic imaging guidance during cannulation and throughout ECMO support are essential to minimize cannulation-related complications and optimize circuit performance.

### 3.6. Surgery

Among the unresolved issues in the management of infants with CDH, the optimal timing of diaphragmatic repair in those requiring ECMO remains one of the most debated topics. Four main surgical strategies have been described: repair before ECMO initiation, early repair during ECMO (generally within the first 72 h after cannulation), late repair during ECMO (performed either shortly before the anticipated decannulation or as a salvage procedure in patients who cannot be successfully weaned), and repair after successful ECMO weaning and decannulation.

Each strategy offers potential advantages and drawbacks. Repair during ECMO may restore normal mediastinal anatomy, improving lung expansion, pulmonary blood flow, venous return, cardiac performance, and gas exchange [64,65,66,67,68]. In addition, maintaining extracorporeal support throughout the immediate postoperative period may facilitate recovery from the transient cardiorespiratory deterioration frequently observed after CDH repair [69,70]. Early intervention may also avoid the technical challenges associated with prolonged ECMO support, such as generalized edema, inflammation, and hepatosplenic enlargement, which increase tissue friability and complicate surgical dissection. Conversely, repair during ECMO is performed under systemic anticoagulation and therefore carries an increased risk of perioperative bleeding. Delaying surgery until after decannulation minimizes this hemorrhagic risk but exposes the patient to the possibility of dying before repair can be performed [19,64,65,68,70,71].

Despite its clinical importance, the optimal timing of repair remains uncertain. Current evidence is derived almost exclusively from retrospective observational studies with heterogeneous patient populations, variable definitions of surgical timing, and inconsistent adjustment for disease severity, limiting direct comparisons and precluding definitive recommendations.

Several registry analyses, including those from the Congenital Diaphragmatic Hernia Study Group [72] and the ELSO Congenital Diaphragmatic Hernia Interest Group [68], together with a recent meta-analysis and multiple single-center studies [70,73,74,75], have reported better survival following repair after successful ECMO decannulation than after repair during ECMO. These studies also describe shorter ECMO duration [70,73,74,76] and, in some series, lower rates of perioperative bleeding [70,73,74], whereas hospital length of stay [70,73] and neurological outcomes [68] appear to be similar between strategies. However, Danzer et al. [77] reported an increased risk of early neurocognitive and neurofunctional impairment among children repaired during ECMO. Overall, these findings should be interpreted with caution because many studies excluded infants who died before surgical repair, introducing substantial survivor bias that may overestimate the apparent benefit of post-decannulation repair.

When repair after successful decannulation has been specifically compared with late repair during ECMO, no study has demonstrated a clear advantage of maintaining extracorporeal support in infants who are otherwise ready for weaning. In a large cohort from the Congenital Diaphragmatic Hernia Study Group [78], only 2.8% of infants repaired after ECMO decannulation required a second ECMO run or died within 72 h after surgery, suggesting that, in this subgroup, the potential benefit of postoperative ECMO support may not outweigh the increased bleeding risk associated with surgery under systemic anticoagulation.

Nevertheless, this comparison does not resolve the main clinical dilemma, as neither the likelihood nor the timing of successful ECMO weaning can be reliably predicted at the onset of extracorporeal support [79,80]. Consequently, the focus has shifted from identifying a universally optimal timing of repair toward determining which patients are most likely to benefit from each surgical strategy.

In this context, increasing interest has centered on early repair during ECMO. A recent meta-analysis [81], several single-center studies [71,82,83], and two registry-based analyses from the Congenital Diaphragmatic Hernia Study Group that accounted for survivor bias suggest a potential benefit of an early repair strategy. In one registry analysis, early repair was associated with improved survival compared with late repair during ECMO [84], whereas the other demonstrated a survival advantage over repair after ECMO decannulation [85]. Some studies have also reported shorter ECMO duration [83,84,86], fewer days of mechanical ventilation [83], and shorter hospital length of stay [83,84] following early repair. Notably, early repair has also been associated with a reduced need for perioperative blood product transfusion [87]. This may be particularly relevant in the ECMO population, as postoperative transfusion—especially platelet transfusion—has been associated with increased mortality among infants with CDH requiring ECMO [88]. Preliminary single-center data [67] further suggest that repair within the first 24 h after ECMO cannulation may achieve outcomes comparable to those reported with an early repair strategy.

Importantly, the favorable outcomes reported with early repair have largely been achieved in centers using standardized perioperative management protocols [71,76,83,86]. Careful adjustment of systemic anticoagulation, close monitoring of coagulation parameters and platelet counts, routine administration of antifibrinolytic agents, and meticulous surgical technique—including gentle tissue handling, meticulous hemostasis, limited dissection of the posterior diaphragmatic rim, and appropriate use of electrocautery and topical hemostatic agents—appear to substantially reduce perioperative bleeding [71,83,86]. In selected patients, a staged strategy consisting of temporary repair during ECMO followed by definitive reconstruction after decannulation has also been proposed to further minimize operative trauma and hemorrhagic complications [79,89].

Finally, Kays et al. [90] proposed that, for the most severe neonates with left-sided CDH and liver herniation, surgical repair during the first hours of life, before the anticipated initiation of ECMO, may represent the optimal strategy to maximize survival.

Taken together, the available evidence suggests that the goal should not be to identify a single optimal timing for diaphragmatic repair but rather to determine which surgical strategy is most appropriate for each individual patient. The timing of repair should therefore probably be individualized according to prenatal and postnatal predictors of disease severity, the anticipated likelihood of successful ECMO weaning, the patient’s clinical evolution and coagulation status, associated comorbidities, and the treating center’s expertise with repair performed during or after ECMO.

### 3.7. Management on ECMO

The management of neonates with CDH on ECMO follows the general principles of neonatal ECMO [91], with particular attention to persistent pulmonary hypertension, right ventricular function, and pulmonary recovery [56]. Lung-protective ventilation with low airway pressures and permissive hypercapnia should be maintained to minimize further injury to the hypoplastic lungs [19,92,93,94].

The optimal duration of ECMO in CDH remains uncertain. Pulmonary hypoplasia and pulmonary hypertension may require prolonged support, particularly in severe CDH. Therefore, CDH is a common diagnosis among neonates requiring prolonged ECMO, defined by Prodhan et al. as support exceeding 21 days in an ELSO registry analysis [95]. Kays et al. reported survival among patients remaining on ECMO of 56%, 46%, and 43% at 2, 3, and 4 weeks, respectively, decreasing to 15% after 5 weeks, with no survivors after 40 days. Nevertheless, these findings should not be interpreted as a fixed threshold for futility, as selected patients may recover after prolonged support. In the same cohort, 7 of 16 patients requiring a second ECMO run survived to discharge [41]. Therefore, decisions regarding continuation or withdrawal of ECMO should be individualized according to pulmonary and cardiac recovery, end-organ function, and the development of ECMO-related complications.

Complications during ECMO support in CDH are frequent. Mechanical complications, including circuit thrombosis, cannula-related problems, and oxygenator dysfunction, occur in a substantial proportion of patients, while renal and hemorrhagic complications are particularly clinically significant [96,97]. Acute kidney injury is common, with rates of up to 71% reported in CDH patients requiring ECMO, and severe renal dysfunction is associated with lower survival and prolonged ECMO support [98,99,100]. Hemolysis may further contribute to renal injury [55,96]. Chylothorax is also an important postoperative complication, reported in 7–28% of CDH patients, and ECMO is an independent risk factor: in a large CDHSG registry cohort, chylothorax developed in 8.6% of ECMO-treated neonates compared with 3.2% of those not treated with ECMO (aOR 2.9), with the highest risk when repair was performed on or after ECMO [101,102]. The risk of bloodstream infection increases with prolonged ECMO duration [103]. Finally, bleeding is another major complication, particularly around the site of surgical repair; major bleeding has been strongly associated with mortality among CDH patients on ECMO [68,88] (Table 2). Balanced anticoagulation in this group of patients is challenging, especially after surgical repair. Recently, bivalirudin has emerged as an agent that may reduce bleeding events [104], although this has not yet been confirmed in larger cohorts [105].

ECMO weaning represents a particularly vulnerable phase, as some patients may deteriorate after decannulation and require a second ECMO run [106]. Given the potential difficulty of repeat cannulation [107], some centers have considered maintaining cannulas in situ for a short period in selected high-risk patients, although evidence supporting this strategy remains limited [41,108,109]. Overall, ECMO management in CDH requires balancing sufficient time for pulmonary and cardiovascular recovery against the cumulative morbidity associated with prolonged extracorporeal support.

### 3.8. Outcomes

Outcomes among infants with CDH receiving ECMO vary substantially across centers. In an analysis of the ELSO registry including 4223 neonates with CDH treated with ECLS across 109 centers between 2000 and 2019, observed mortality varied markedly between centers, ranging from 14.3% to 90.9%, with an interquartile range of 42.9–62.1% [110]. After adjustment for pre-ECLS case mix, 13 centers had significantly better standardized mortality ratios, and 7 had significantly worse standardized mortality ratios than the median, whereas the majority did not differ significantly from the median [110]. These findings highlight the potential influence of patient selection, institutional experience, and center-specific management strategies on outcomes and suggest that variation in ECLS outcomes cannot be fully explained by differences in pre-ECLS case mix.

Although survival of neonates with severe CDH has improved, survivors remain at risk of long-term multisystem morbidity, and neurodevelopmental impairment (NDI) is among the most relevant. An early UK cohort of 73 neonates treated with ECMO reported 58% survival to hospital discharge and 37% survival at 1 year; among the 27 long-term survivors, 13 had respiratory symptoms, 16 had gastrointestinal problems, and 7 had significant neurodevelopmental problems, although no formal neurodevelopmental testing was performed and no non-ECMO comparison group was included [111]. In a systematic review of 33 studies including 3541 CDH survivors, NDI was reported in 23%, and a meta-analysis of the three available comparative studies showed that NDI was more frequent among ECMO-treated survivors than among non-ECMO survivors (39/80, 49% vs. 48/216, 22%; RR 2.27, 95% CI 1.63–3.17) [112]. However, the contribution of ECMO itself to NDI remains controversial. Randomized evidence is not available to resolve this question: in the only randomized trial of neonatal ECMO, all 17 infants with CDH allocated to conventional management died, compared with 14 of the 18 allocated to ECMO [16], and at 4 years of age 16 of the 18 infants allocated to ECMO had died or survived with severe disability [17], precluding any comparison of neurodevelopmental outcomes between treatment strategies in CDH. Consequently, the available evidence is observational. The above meta-analysis was based on a small number of unadjusted studies, and the quality of evidence was rated as low because of possible confounding and imprecision; its authors acknowledged that the causative role of ECMO is difficult to establish [112]. Moreover, because ECMO is reserved for the most severely affected infants, ECMO exposure is closely linked to disease severity, including pulmonary hypoplasia, pulmonary hypertension, pre-cannulation hypoxemia and hemodynamic instability, and longer duration of mechanical ventilation and hospital stay, which makes disentangling the effect of extracorporeal support from that of the underlying illness challenging. Observational cohorts have yielded conflicting results. Some studies have reported poorer outcomes among ECMO-treated children: in a large single-center cohort of 212 CDH survivors assessed at a median age of 22 months, the 50 ECMO-treated children scored lower on Bayley-III cognitive and motor composites, particularly those repaired on ECMO; however, the authors could not control for confounders and acknowledged that it remains unknown whether these deficits reflect disease severity, the treatment itself, or both [77]. At 8 years of age, ECMO-treated survivors in a Dutch cohort (n = 16) had a lower mean IQ than non-ECMO survivors (n = 19; 91.7 vs. 111.6), whereas motor and concentration problems were frequent in both groups without significant differences between them; the authors attributed the poorer outcome mainly to the severity of illness necessitating ECMO rather than to ECMO itself [113]. Motor difficulties persisted up to 12 years of age, mainly among ECMO-treated children [114]. More recently, a prospective longitudinal cohort of 278 children (130 treated with ECMO) followed up to 6 years of age found that cognitive delays were more frequent in the ECMO group, whereas language and motor outcomes were similar and most delays were mild. In multivariable analysis, ECMO was independently associated with cognitive delay only at 6 months of age (OR 2.5, 95% CI 1.1–5.5), whereas at later ages markers of disease severity and clinical course, such as liver herniation, patch repair, duration of mechanical ventilation, and reoperation within the first year, were the independent predictors; most children had no delay in any domain by 6 years of age [115]. Similarly, in a Japanese multicenter cohort of 132 children with isolated CDH, the observed-to-expected lung-to-head ratio was the only independent predictor of NDI at 3 years, whereas ECMO did not reach statistical significance (adjusted OR 4.17, 95% CI 0.33–52.43), although only six children had received ECMO [116]. Neurodevelopmental difficulties have also been reported in a centre where ECMO is rarely used (1 of 83 children), with language impairment in 23% of children at 2 years and verbal intellectual impairment in 25% at 8 years, supporting the view that CDH and its perinatal course confer neurodevelopmental risk independently of extracorporeal support [117]. Overall, the available evidence does not allow the effect of ECMO to be separated from that of disease severity, and ECMO should be regarded, at least in part, as a marker of a high-risk population rather than as an established independent cause of NDI. Whether specific ECMO-related factors, such as intracranial hemorrhage or repair on ECMO [68], add to this risk should be addressed in prospective multicentre studies with standardized assessments and adjustment for disease severity. Finally, neuropsychological outcome may appear normal at preschool age, with deficits becoming apparent only once higher cognitive functions are demanded at school age [118].

Beyond neurodevelopment, CDH survivors are at risk of respiratory, nutritional, gastrointestinal, and musculoskeletal morbidity, particularly after severe disease requiring ECMO, patch repair, or prolonged respiratory support [119]. Data from FETO-treated survivors further suggest that these postnatal severity markers, rather than prenatal therapy history, are associated with respiratory and neurological outcomes at 2 years [120]. Long-term multidisciplinary follow-up should therefore be an integral component of CDH care, with its intensity tailored to the severity of the neonatal course.

## 4. Conclusions

**Figure 2:**
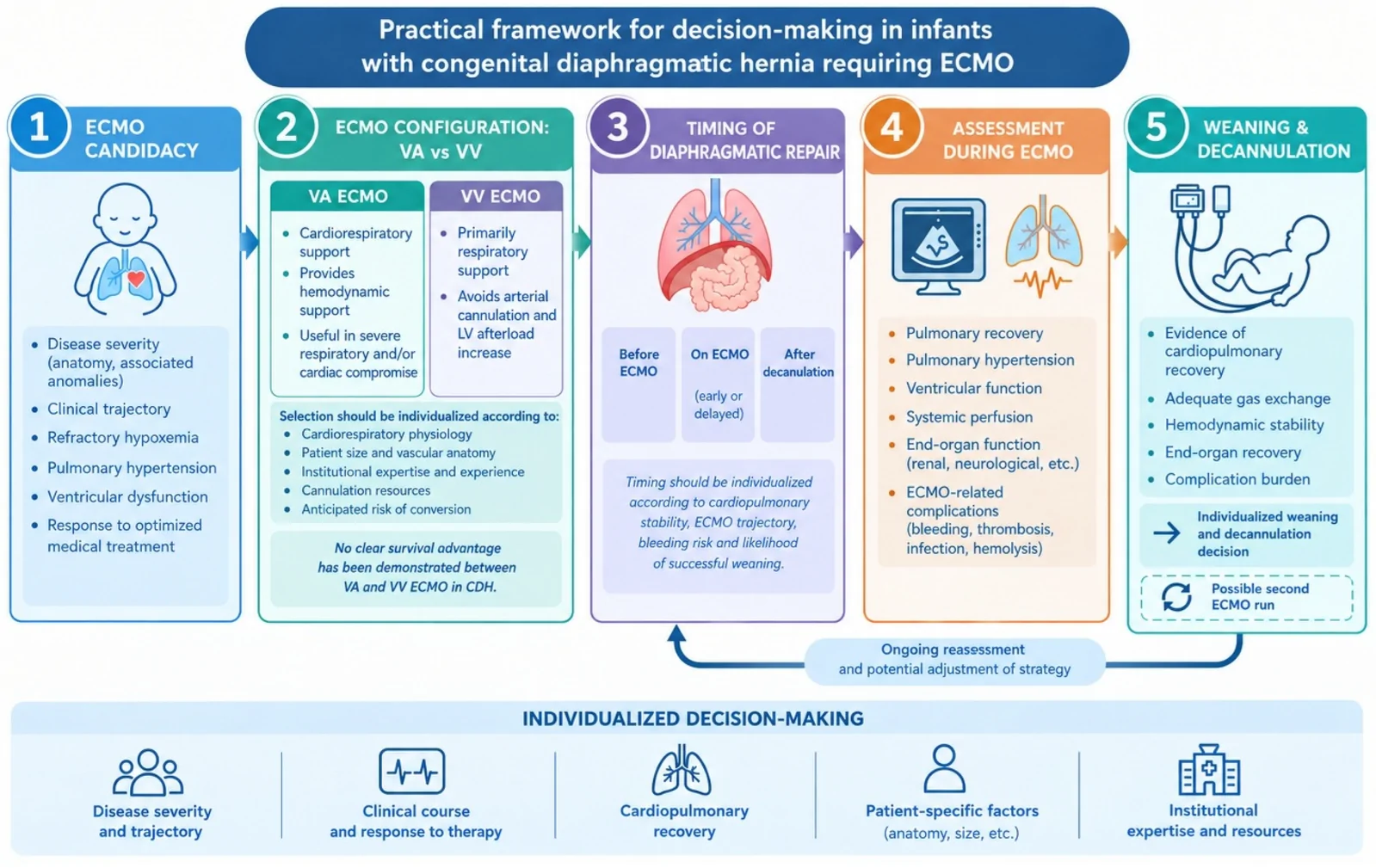
Practical framework for decision-making in infants with congenital diaphragmatic hernia requiring ECMO. The figure summarizes the major clinical decision points discussed in this review. ECMO candidacy, mode selection, timing of diaphragmatic repair, assessment during ECMO, and weaning and decannulation should be individualized according to disease severity, clinical trajectory, cardiopulmonary recovery, patient-specific factors, and institutional expertise. The framework is not intended as a validated clinical algorithm.

Management of infants with CDH requiring ECMO remains an area of substantial clinical uncertainty. Current evidence is derived predominantly from retrospective, single-center, and registry-based studies, with limited randomized evidence and substantial heterogeneity in study populations and management strategies. Across the major domains addressed in this review—including ECMO candidacy, cannulation strategy, mode of support, surgical timing, and duration of ECMO—no single physiological threshold or universal protocol has been shown to reliably predict outcome or define the optimal approach. Instead, the available evidence supports an individualized strategy that integrates prenatal and postnatal markers of disease severity, clinical trajectory, cardiopulmonary recovery, and institutional expertise (Figure 2).

Future prospective, multicenter studies using standardized definitions and adequately accounting for disease severity will be essential to refine risk stratification, strengthen the evidence base for key management decisions, and reduce the substantial practice variation that continues to characterize the care of this high-risk population.

## Figures and Tables

**Table 1:** Criteria proposed for initiating extracorporeal life support (ECLS) in congenital diaphragmatic hernia (CDH): ELSO interim guidelines vs. CDH EURO Consortium consensus.

Domain	ELSO Interim Guidelines (Guner 2021) [19]	CDH EURO Consortium 2015 Update (Snoek 2016) [30]
Oxygenation index	Not specified	OI ≥ 40 present for at least 3 h
Oxygenation	Inability to achieve or maintain preductal SpO_2_ > 85%	Inability to maintain preductal SpO_2_ > 85% or postductal SpO_2_ > 70%
Ventilator settings	CMV: PIP > 26–28 cmH_2_O, PEEP > 6 cmH_2_O, RR > 50/min. HFOV: MAP > 14 cmH_2_O, frequency < 7 Hz, amplitude > 40	PIP > 28 cmH_2_O or MAP > 17 cmH_2_O required to achieve saturation > 85%
Ventilation/acidosis	Persistent severe respiratory acidosis (PCO_2_ > 70 mmHg) with pH < 7.20	Increased PaCO_2_ and respiratory acidosis with pH < 7.15 despite optimization of ventilator management
Oxygen delivery	Inadequate DO_2_ with metabolic acidosis (no thresholds)	Inadequate DO_2_ with metabolic acidosis: lactate ≥ 5 mmol/L and pH < 7.15
Perfusion/hemodynamics	Inadequate end-organ perfusion (lactate > 3 mmol/L, oliguria); systemic hypotension refractory to fluids and vasoactive drugs; hemodynamic instability refractory to inotropes/chronotropes	Systemic hypotension resistant to fluid and inotropic therapy, with urine output < 0.5 mL/kg/h for at least 12–24 h
Cardiac/pulmonary vascular	Pulmonary hypertension ± RV dysfunction; left ventricular failure	Not specified
Acute deterioration	Preductal SpO_2_ < 70% not recovering after ventilator optimization	Not specified

Note: Adapted from Guner 2021 and Snoek 2016. No uniformly accepted or rigidly applied criteria exist for ECLS initiation in CDH [19]; the ELSO column summarizes expert-opinion consensus indications. CDH EURO criteria are grade D recommendations. Broadly accepted patient selection criteria are GA ≥34 weeks and birth weight >2 kg. CMV, conventional mechanical ventilation; HFOV, high-frequency oscillatory ventilation; PIP, peak inspiratory pressure; PEEP, positive end-expiratory pressure; RR, respiratory rate; MAP, mean airway pressure; OI, oxygenation index; DO_2_, oxygen delivery; RV, right ventricular; SpO_2_, peripheral oxygen saturation.

**Table 2:** Most frequent complications reported in neonates with congenital diaphragmatic hernia (CDH) treated with extracorporeal membrane oxygenation (ECMO), drawn from single-center series, multicenter cohorts, and ELSO registry analyses. Reported incidences vary across studies due to differences in patient selection, era, and complication definitions. Only data from CDH patients treated with ECMO are shown; for cohorts that also included patients not treated with ECMO, the ECMO subgroup is reported. Study design, period, and sample size are given for each estimate, with numerators and denominators (*n/N*) where reported.

Complication	Reported Incidence/Key Finding	Design, Period and Sample Size	Reference
Any complication (overall)	38/54 (70.4%) had ≥1 complication; survival 33/54 (61.1%). Mechanical (12/21 vs. 9/33) and renal (6/21 vs. 0/33) complications more frequent in non-survivors than in survivors	Single center, retrospective (USA), 2005–2020; N = 54	Stewart et al. 2022 [97]
Acute kidney injury (AKI)	AKI (RIFLE) in 48/68 (71%); “failure” stage in 33/68 (49%), with survival of 9/33 (27.3%) vs. 28/35 (80%) without failure and longer ECLS duration (314 vs. 197 h); CRRT in 11/68 (16%)	Single center, retrospective (USA), 1999–2009; N = 68	Gadepalli et al. 2011 [99]
	AKI (KDIGO) in 15/24 (62%) ECMO-treated neonates, stage 3 in 12 of them; CRRT required during ECMO in 12/24	Single center, retrospective (Sweden), 2015–2022; ECMO subgroup, n = 24 (of 109 CDH neonates)	Andersson et al. 2025 [98]
	AKI (neonatal modified KDIGO) in 26/32 (81%) ECLS-treated neonates; survival 35% with AKI vs. 100% without; plasma-free hemoglobin >50 mg/dL associated with AKI	Single center, retrospective (USA), 2009–2017; ECLS subgroup, n = 32 (of 90 CDH neonates)	Liberio et al. 2021 [100]
Hemolysis	Hemolysis in 14/62 (22.6%); mortality 14/14 (100%) with hemolysis vs. 15/48 (31.1%) without; more frequent with 8 Fr than with 13 Fr cannulae (43% vs. 17%)	VV-ECMO; single center, prospective (Germany), 2013–2020; N = 62	Lemloh et al. 2023 [55]
	Hemolysis (plasma-free Hb >50 mg/dL) in 479/4576 (10.5%); mortality 66% with hemolysis vs. 50.4% without hemolysis; centrifugal pump strongest risk factor (OR 6.67, 95% CI 5.14–8.67)	ELSO registry, multicenter, 2000–2015; N = 4576	Guner et al. 2021 [96]
Chylothorax	Chylothorax in 33/382 (8.6%) ECMO-treated neonates vs. 32/1001 (3.2%) without ECMO ^‡^; ECMO independently associated with chylothorax (aOR 2.9, 95% CI 1.7–4.7), with the highest risk after repair on ECMO (aOR 2.6) or after ECMO (aOR 3.1)	CDHSG registry, multicenter, 2007–2010; repaired CDH; ECMO subgroup, n = 382 (of 1383)	Levy et al. 2013 [102]
Bloodstream infection (BSI)	18/338 (5.3%) had ≥1 confirmed BSI; risk increased with ECMO duration (2/315, 0.6%, in week 1 vs. 5/58, 8.6%, after 21 days)	Children’s Hospitals Neonatal Database, multicentre (USA), 2010–2016; N = 338	Keene et al. 2019 [103]
Intracranial hemorrhage/severe neurologic injury *	Hemorrhagic complications in 12/54 (22.2%) and neurological complications in 10/54 (18.5%)	Single centre, retrospective (USA), 2005–2020; N = 54	Stewart et al. 2022 [97]
	Severe neurologic injury in 12.6% after on-ECMO repair vs. 8.7% after repair post-decannulation (unmatched); higher odds with on-ECMO repair in the matched cohort (OR 1.49, 95% CI 1.13–1.96), not significant after adjustment for ECMO duration (OR 1.33, 95% CI 0.99–1.79)	ELSO registry, multicentre, 2000–2016; N = 3045 repaired on ECMO (n = 1817) or after ECMO (n = 1228); propensity-matched, N = 2224 (1112 per group)	Delaplain et al. 2019 [68]

* Severe neurologic injury: CNS hemorrhage, infarct, and/or intraventricular hemorrhage (IVH) grade 3 and 4. ^‡^ Numbers calculated from the percentages reported in the original article. Estimates derived from small, single-center cohorts (e.g., mortality among the 14 neonates with hemolysis in Lemloh et al.) should be interpreted with caution and do not represent a generalizable risk. N, total study cohort; n, number of patients in the subgroup or with the event; aOR, adjusted odds ratio; Hb, hemoglobin; Fr, French. CDH, congenital diaphragmatic hernia; ECMO, extracorporeal membrane oxygenation; ECLS, extracorporeal life support; ELSO, Extracorporeal Life Support Organization; VV, veno-venous; AKI, acute kidney injury; RIFLE, Risk, Injury, Failure, Loss of kidney function, End-stage renal disease (classification); KDIGO, Kidney Disease: Improving Global Outcomes; CRRT, continuous renal replacement therapy; CDHSG, Congenital Diaphragmatic Hernia Study Group; OR, odds ratio; CI, confidence interval; CNS, central nervous system.

## Data Availability

No new data were created or analyzed in this review. Data sharing is not applicable to this review.
